# Movement Time of Lower Trunk Muscles during Dynamic Postural Control in Response to a Sudden Visual Stimulus during Walking: A Pilot Study

**DOI:** 10.3390/ijerph18095015

**Published:** 2021-05-10

**Authors:** Jaehyun Jung, Kewwan Kim, Sungjae Choi, Gwangyu Song, Young Ryu, Cholhee Kim, Chaegil Lim

**Affiliations:** 1 College of Medicine, Korea University, Seoul 02841, Korea; mcfriend82@naver.com (J.J.); csjmd888@korea.ac.kr (S.C.); gsong@kumc.or.kr (G.S.); 2 Division of Rheumatology, Department of Internal Medicine, Korea University Ansan Hospital, Ansan 15355, Korea; 3 Department of Physical Education, College of Arts and Physical Education, Incheon National University, Incheon 22012, Korea; kewwan09@incheon.ac.kr; 4 Department of Internal Medicine, Division of Rheumatology, Korea University Guro Hospital, Seoul 08308, Korea; 5 Department of Physical Education, Graduate School of Incheon National University, Incheon 22012, Korea; gym04@hanmail.net; 6 Department of Physical Therapy, Gachon University, Incheon 21936, Korea

**Keywords:** dynamic postural control, walking, movement time, visual stimuli, lower trunk muscle

## Abstract

Postural control during walking is maintained by the combination of various factors. Among these factors, adjustment of trunk movement is essential for maintaining postural control, and the response of muscles to unpredictable stimuli affects postural control. Loss of balance while walking increases the risk of accidents, the frequency of which depends on age and sex. In this study, we investigated whether there was a difference in the movement time of trunk muscles to sudden stimulation while walking according to age and sex. Fourteen healthy individuals aged 20–30 years (6 men, 8 women) and 12 individuals aged 50–70 years (4 men, 8 women) were included in the study. Movement time of bilateral erector spinae and rectus abdominis muscles in response to visual stimulation during walking was examined using surface electromyography. Movement time was calculated as the total muscle activation time excluding the reaction time. This study revealed no significant differences in movement time of the erector spinae muscles according to sex or age. The role of the rectus abdominis muscles in maintaining posture during walking was insignificant. In conclusion, the movement time of trunk muscles in response to sudden visual stimulation during walking did not differ by age or sex, and the difference in accident frequency may be associated with deterioration of other factors required to maintain posture.

## 1. Introduction

Many different forms of information affect walking style. The human body is able to walk when various factors, such as the musculoskeletal and nervous systems, work together in harmony via integrated information processing [1]. Balance is essential for walking, and vision, vestibular sense, proprioception, and muscle strength are important for maintaining balance [2]. During such a complex process, if an element’s function becomes impaired, accidents, such as falling, may occur. The fall risk in older adult is high, which is attributed to decreased vestibular or visual function [3,4]. Vestibular organ function is decreased in more than 30% of individuals aged 50 years and older, and vision, including visual processes such as visual activity, contrast sensitivity and accommodation, are significantly lower in individuals aged 50 years and older, compared to that in younger people [2,4].

Controlling trunk movement is essential for balance maintenance [5]. During ambulation, trunk muscles work synergistically to maintain postural control. Both the abdominal muscles, including the rectus abdominis and external oblique muscles, and the spine extensor muscles, including the erector spinae and multifidus muscles, work to control ambulatory posture. Among the trunk muscles, the rectus abdominis plays an important role in balance, along with the erector spinae and multifidus muscle [6]. As age increases, trunk muscles weaken and lose function, which is a risk factor for accidents such as falls [7]. However, while the degradation of visual processes or vestibular function is difficult to recover, muscle strength or function can be increased through exercise. In maintaining posture during ambulation, if one function deteriorates, others compensate to attempt correction of the defect [1].

To maintain stable posture with sudden changes from a stimulus, both the strength of the muscle and reaction speed are critical. Strengthening the muscles helps maintain posture and allows the muscles to properly move in response to the stimulus [7]. In older adult individuals, muscle strength and function weaken from oxidative stress [8]. In addition, due to differences in muscle metabolism between men and women, muscle strength and function are also different [9]. The causes of falls are many, but a higher frequency of falls in women than in men may be due to differences in muscle metabolism [10,11]. Trunk muscles are crucial for the maintenance of body balance and postural control; therefore, investigating which muscle function declines with regard to age and sex in response to stimulation is useful information for exercise prescription.

Of all the human sensory organs, vision provides the most information and is an essential element of postural maintenance and control [12]. A change in visual information has a significant effect on the maintenance and adjustment of posture, which is essential to walking. The purpose of this study was to determine how quickly the trunk responds to a sudden visual stimulus during ambulation. Specifically, we measured the movement time excluding the reaction time during muscle activity and compared the differences in movement time according to age and sex.

## 2. Materials and Methods

### 2.1. Participants

Participants were selected via open recruitment at an orthopedic clinic in Korea from June 2018 to April 2019. Participants were aged 20–30 and 50–70 years and had no visual deficits; no history of musculoskeletal disorders such as fractures, arthritis, and myopathy; and no history of neurologic disorders such as stroke, neuropathy, and dementia were recruited. All volunteers underwent a skeletal survey and visual acuity test, and participants with no radiologic abnormality and corrected vision 0.8 and higher were included in the study. A total of 14 individuals aged 20–30 years (six men, eight women) and 12 aged 50–70 years (four men, eight women) were included in this study. For the main trial, which was designed with 90% power and two-sided 5% significance, we recommended pilot trial sample sizes per treatment arm of 75, 25, 15 and 10 for standardized effect sizes that are extra small (≤0.1), small (0.2), medium (0.5) or large (0.8), respectively [13]. On assessment day, participants were asked not to exercise or to consume caffeinated drinks. All study participants provided informed consent, and the study was approved by Incheon National University (IRB 7007971-201804-002(01~02)). This study was conducted in accordance with the Helsinki Declaration, 2000.

### 2.2. Main Variables and Covariates

**Figure 1:**
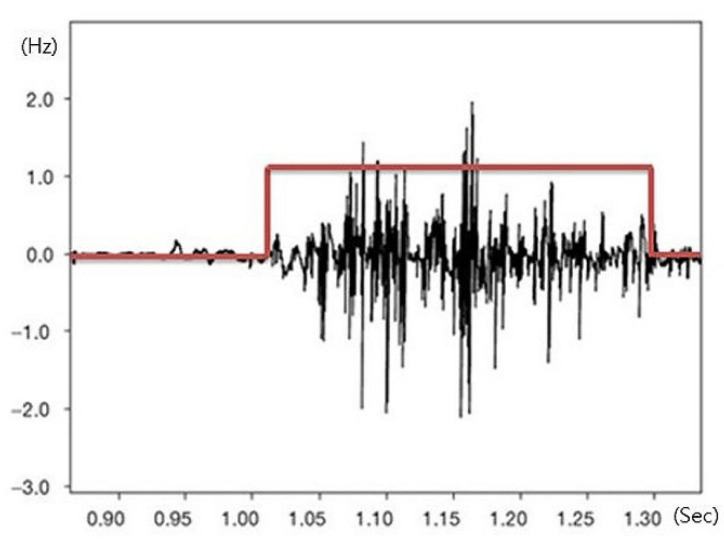
Signal of movement time.

**Figure 2:**
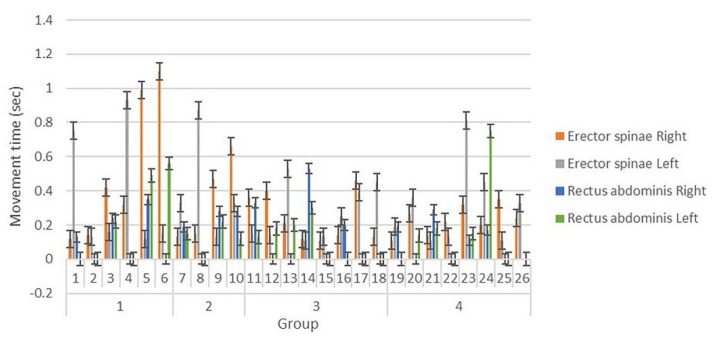
Movement time.

Movement times of the left and right erector spinae muscles and rectus abdominis muscles were measured as indicators of dynamic postural control of the lower trunk during sudden visual stimulation. Onset and offset times of the muscles were measured based on the intervals between the delivery of visual information, and these data were used to calculate movement time for postural control. Movement time of these muscles was recorded using 4-channel surface electromyography (sEMG) (Telemyo DTS & DTS EMG, Noraxon Inc., Scottsdale, AZ, USA). The time intervals of measured muscle activity were analyzed using the MyoResearch XP software (ver. 1.08.17, Noraxon Inc., Scottsdale, AZ, USA). The sampling rate of the sEMG signals was 1500 Hz, and a 10–500 Hz band-pass filter was used alongside a 60 Hz notch filter. Raw data delivered from the muscle were quantified as the root mean square. To prevent a learning effect, participants were allowed one trial in an independent space after viewing an example of the measurement method. Truncal hair was shaved, and the top layer of skin was removed with sandpaper. The exposed layer was wiped with an alcohol swab to minimize noise generation. Electrodes were attached according to the sEMG for non-invasive assessment of muscles (SENIAM) guidelines [14]. After the electrodes were applied to the rectus abdominis (2 cm lateral from the midline of the umbilicus) and erector spinae (2 cm lateral to the 3 lumbar spine vertebra level), participants walked on the flat treadmill at a speed similar to their normal walking pace. A sling was used to prevent falls. In front of the treadmill, a monitor was installed at eye level so participants could receive sudden visual stimuli, which was a video of a car suddenly rushing to the front of a participant in the opposite lane, while walking. A blackout curtain was suspended around the treadmill to prevent external visual stimuli. Auditory stimuli were blocked with earplugs. A lamp illuminating the experimental set-up was extinguished at the start of the procedure so that participants could concentrate on the images shown on the monitor. Participants were allowed one minute to adjust to the treadmill, after which the measurement was carried out. Visual information was provided 60 s after the participants started walking, and the visual playback lasted 20 s. Sudden visual stimuli occurred for 2 s at the playback midpoint (Figure 1 and Figure 2).

Sex, age, and body mass index (BMI) were potential confounding variables affecting movement time [4,15,16]. Participants were divided into four groups according to sex and age. Group 1 included 20–30-year-old men, Group 2 included 50–70-year-old men, Group 3 included 20–30-year-old women, and Group 4 included 50–70-year-old women. BMI was calculated by dividing weight (kg) by the square of height (m) and adjusted for analysis when comparing movement time between groups.

### 2.3. Statistical Analysis

Statistical analysis of the experimental data was performed using SPSS Statistics 23.0 for Microsoft Windows (SPSS Inc., Chicago, IL, USA). The means and standard deviations (SD) of movement time were calculated using descriptive statistics. For continuous variables, the Kruskal–Wallis H test was used, and an analysis of covariance (ANCOVA) was performed for comparing movement time in each group after adjusting for BMI. Moreover, a partial eta squared (η^2^) was used to explore the effect size. All *p* values were two-tailed, and *p* < 0.05 was considered statistically significant.

## 3. Results

### 3.1. Characteristics of Participants

The mean age in each group was as follows: Group 1, 25.3 ± 1.4 years; Group 2, 58.5 ± 6.9 years; Group 3, 25.9 ± 2.2 years; and Group 4, 58.6 ± 5.4 years. Both men and women were taller in the younger age groups; however, weight was higher in younger men and in older women. There were also significant differences in BMI between the four groups (Table 1).

### 3.2. Differences in Movement Time by Sex and Age

Movement time of the erector spinae and rectus abdominis muscles is shown in Table 2. Average movement time of the erector spinae muscles was longer in men than in women. The data for movement time of the rectus abdominis muscles had many missing values and thus, were not analyzed between groups. Missing values were muscle movements not detected by the sEMG, which suggests that the muscle was inactive or less active during the movement. There was no significant difference in the movement time of erector spinae muscles according to sex and age after adjustment for BMI (Table 3 and Table 4).

## 4. Discussion

Movement time of the erector spinae and rectus abdominis was assessed during ambulation when sudden visual stimuli was introduced. No differences in movement time of these muscle groups were noted once BMI was corrected for age and sex. This suggests that the difference in postural control according to age or sex is due to reasons not related to the trunk muscles, and deterioration of trunk muscles do not cause falls.

In the older age groups, weakness of trunk extensors is a cause of the decrease in static balance ability [17]. In this study, there was no difference in the response time of erector spinae with age. This suggests that muscle weakness is not significantly related to a decrease in muscle reaction velocity; rather, muscle weakness is associated with a decrease in muscle mass seen in older people, especially in women who are postmenopausal [18]. In addition, not only the trunk muscles but also the muscles of the lower extremities at the ankles, knees, and hips are used to maintain balance [19,20]. Lower extremity muscle weakness is related to motor conduction velocity or compound motor action potential amplitude [21], and a decrease in the reaction time of the lower extremity muscles may be associated with a high fall rate in older adult people. Fabio V. dos Anjos et al. found that medial gastrocnemius was more active in the aged (74.1–98.2%) than the young (interquartile interval, 44.9–81.9%) for longer periods of time and the tibialis anterior muscle was also more active in the aged (2.6–82.5%) than the young (0.7–4.4%) [22]. Moreover, Monika Błaszczyszyn et al. reported tibialis anterior, peroneus, gastrocnemius medialis, and gastrocnemius lateralis of young and old women who were measured. All muscles except the gastrocnemius medialis muscle showed higher muscle activity in old rather than young women [23]. For that reason, older people control their posture by using more calf muscles than younger people to prevent falls. In clinical practice, it is important to secure static and dynamic stability through the use of trunk muscles when gait training is performed to prevent falls, but more focus should be placed on securing stability through the lower leg muscles.

Women have less strength and slower contraction velocity in lower extremity muscles than men, and their risk of falls or injuries is greater [11,24]. Hormonal action and mitochondrial differences affect muscle metabolism, resulting in differences in muscle strength and velocity, which is a cause of increased muscle fatigue in women [9,25]. However, in this study, no significant differences were found in the velocity of specific trunk muscle contractility between men and women of any age. To maintain balance, stability of the trunk muscles is essential, and the size, strength, and activity of the muscles are all involved in stability [25,26]. The trunk muscles of men are larger and not as easily fatigued as those of women, which affects the incidence of falls. In this study, muscle mass was not measured, and only specific trunk muscles were analyzed. However, that the muscle mass of the trunk is related to truncal reaction time is still insufficient evidence, and synchronization of the lower extremity muscles with trunk muscles is important for maintaining balance. In this study, the muscle reaction time of the trunk muscles was not significantly involved in maintaining balance.

In this study, the movement time of the rectus abdominis in multiple participants was not measured due to missing values from subthreshold activation. To maintain balance, the trunk extensors play a larger role; the role of flexor is small [17], which may be a reason for similar reactions in upper limb muscles during sudden change while walking.

This study stratified sex and age into groups and compared movement time of trunk muscles between groups, but adjusted for potential confounding factors such as sex and age. In maintaining posture, both quantitative and qualitative factors of muscles are important. Sarcopenia diminishes walking speed due to a decrease in muscle strength, and postural control deteriorates [27]. Although there is no information about muscle characteristics other than movement time, BMI, which affects muscle power, was age and sex-adjusted in this study [28].

## 5. Limitation

This study has limitations. First, because sample size is small, the results may differ from those of a similar study with a larger sample size. So this study was marked as a pilot study. Second, movement times of muscles other than the rectus abdominis and erector spinae were not measured. Third, muscle mass or strength was not measured. Finally, we did not assess hypotension, drug use, smoking, and cognitive impairments, which could have affected muscle contraction and postural balance [29].

## 6. Conclusions

In maintaining postural balance against sudden stimulation while walking, there was no significant difference in the movement time of the erector spinae (trunk extensor) according to sex and age. Rectus abdominis (trunk flexor) did not play an essential role in maintaining posture while walking. Since the sample size is small and various factors in addition to movement time of trunk muscles affect balance, further research is needed on this topic.

## Figures and Tables

**Table 1:** Characteristics of individual study participants.

Group	No.	Age (Years)	Height (cm)	Weight (kg)	BMI
1	1	27	176	81	26.2
	2	27	185	82	24.0
	3	25	172	73	24.7
	4	25	182	76	22.9
	5	24	174	72	23.8
	6	24	177	77	24.6
	Mean ± SD	25.3 ± 1.4	177.7 ± 4.9	76.8 ± 4.1	24.35 ± 1.08
2	7	67	171	71	24.3
	8	61	172	64	21.6
	9	54	176	87	28.1
	10	52	165	63	23.1
	Mean ± SD	58.5 ± 6.9	171.0 ± 4.6	71.3 ± 11.1	24.29 ± 2.76
3	11	29	164	45	16.7
	12	28	164	64	23.8
	13	27	158	57	22.8
	14	26	165	52	19.1
	15	26	168	57	20.2
	16	25	157	59	23.9
	17	23	170	57	19.7
	18	23	168	56	19.8
	Mean ± SD	25.9 ± 2.2	164.3 ± 4.7	55.9 ± 5.5	20.77 ± 2.53
4	19	68	151	49	21.5
	20	63	150	45	20.0
	21	61	165	61	22.4
	22	58	165	60	22.0
	23	58	160	52	20.3
	24	57	155	54	22.5
	25	53	156	65	26.7
	26	51	162	70	26.7
	Mean ± SD	58.6 ± 5.4	158.0 ± 5.9	57.0 ± 8.5	22.76 ± 2.59
	*p* *		0.000	0.001	0.035

BMI, body mass index; SD, standard deviation. * *p* values between four groups using Kruskal–Wallis H test.

**Table 2:** Movement time of each participants’ trunk muscles.

Group	No.	Erector Spinae		Rectus Abdominis	
		Right	Left	Right	Left
1	1	0.12	0.75	0.13	-
	2	0.14	0.13	-	-
	3	0.42	0.16	0.24	0.22
	4	0.32	0.93	-	-
	5	0.99	0.12	0.35	0.49
	6	1.10	0.15	-	0.56
2	7	0.13	0.33	0.19	0.15
	8	0.15	0.87	-	-
	9	0.47	0.13	0.28	0.22
	10	0.66	0.33	0.28	0.12
3	11	0.36	0.15	0.33	0.13
	12	0.40	0.14	-	0.18
	13	0.21	0.53	-	0.20
	14	0.12	0.11	0.53	0.30
	15	0.11	0.13	-	-
	16	0.14	0.25	0.20	-
	17	0.46	0.39	-	-
	18	0.13	0.45	-	-
4	19	0.11	0.19	0.19	-
	20	0.27	0.36	-	0.14
	21	0.14	0.11	0.29	0.18
	22	0.22	0.13	-	-
	23	0.32	0.81	0.11	0.15
	24	0.20	0.45	0.17	0.75
	25	0.35	0.11	-	-
	26	0.24	0.33		-

Values are expressed in seconds. SD, standard deviation.

**Table 3:** Comparison of the movement time of erector spinae muscles between groups.

	*p*	
	Right	Left
Group 1 vs. Group 2	0.324	0.821
Group 3 vs. Group 4	0.910	0.503
Group 1 vs. Group 3	0.108	0.238
Group 2 vs. Group 4	0.465	0.390

Group 1: 20–30 year old male; Group 2: 50–70 year old male; Group 3: 20–30 year old female; Group 4: 50–70 year old female.

**Table 4:** Comparison of effect size between groups.

		Group 1	Group 2	Group 3	Group 4	*p*	E(η^2^)
Movement time (s)	ES Rt.	0.52 ± 0.43	0.35 ± 0.26	0.24 ± 0.14	0.23 ± 0.08	0.152	0.210
	ES Lt.	0.37 ± 0.37	0.42 ± 0.32	0.27 ± 0.17	0.31 ± 0.24	0.795	0.045

Values are expressed as mean ± standard deviation; ES: Erector spinae muscle; E, effect size. Group 1: 20–30 year old male; Group 2: 50–70 year old male; Group 3: 20–30 year old female; Group 4: 50–70 year old female.

## Data Availability

The study data are available upon request from the corresponding author. The data are not publicly available because of privacy or ethical restrictions.
